# Peach MYB7 activates transcription of the proanthocyanidin pathway gene encoding leucoanthocyanidin reductase, but not anthocyanidin reductase

**DOI:** 10.3389/fpls.2015.00908

**Published:** 2015-10-26

**Authors:** Hui Zhou, Kui Lin-Wang, Liao Liao, Chao Gu, Ziqi Lu, Andrew C. Allan, Yuepeng Han

**Affiliations:** ^1^Key Laboratory of Plant Germplasm Enhancement and Specialty Agriculture, Wuhan Botanical Garden of the Chinese Academy of SciencesWuhan, China; ^2^Graduate University of Chinese Academy of SciencesBeijing, China; ^3^The New Zealand Institute for Plant & Food Research Ltd., Mt Albert Research CentreAuckland, New Zealand; ^4^School of Biological Sciences, University of AucklandAuckland, New Zealand

**Keywords:** peach, *PpMYB7*, leucoanthocyanidin reductase, anthocyanidin reductase, proanthocyanidins

## Abstract

Proanthocyanidins (PAs) are a group of natural phenolic compounds that have a great effect on both flavor and nutritious value of fruit. It has been shown that PA synthesis is regulated by R2R3-MYB transcription factors (TFs) via activation of PA-specific pathway genes encoding leucoanthocyanidin reductase and anthocyanidin reductase. Here, we report the isolation and characterization of a *MYB* gene designated *PpMYB7* in peach. The peach *PpMYB7* represents a new group of R2R3-MYB genes regulating PA synthesis in plants. It is able to activate transcription of *PpLAR1* but not *PpANR*, and has a broader selection of potential bHLH partners compared with PpMYBPA1. Transcription of *PpMYB7* can be activated by the peach basic leucine-zipper 5 TF (PpbZIP5) via response to ABA. Our study suggests a transcriptional network regulating PA synthesis in peach, with the results aiding the understanding of the functional divergence between R2R3-MYB TFs in plants.

## Introduction

Proanthocyanidins (PAs), also named condensed tannins, are oligomers and polymers of flavan-3-ols, synthesized via the flavonoid pathway. PAs are widely distributed in the plant kingdom, appearing in flowers, fruits, stems, leaves, roots where they provide protection against predation (Mouradov and Spangenberg, 2014). PAs possess strong antioxidant properties (Dixon et al., 2005), and contribute to the prevention of age-related disorders such as cardiovascular diseases, obesity, and cancer (Chaturvedula and Prakash, 2011). However, PAs are also the main cause of astringency in fruits and beverages such as wine, fruit juice, tea, and beer. For example, persimmon accumulates a large amount of PAs in fresh fruits, which causes the bitter and astringent taste and negatively impacts the overall organoleptic quality. Many astringent fruits are not suitable for eating and need an artificial treatment to remove astringency by carbon dioxide and/or ethanol after harvest (Dixon et al., 2013). Fruits are the major dietary source of PAs. To facilitate the genetic improvement of nutritional value and organoleptic quality of fruits, it is necessary to elucidate the complicated network controlling PA biosynthesis.

**FIGURE 1:**
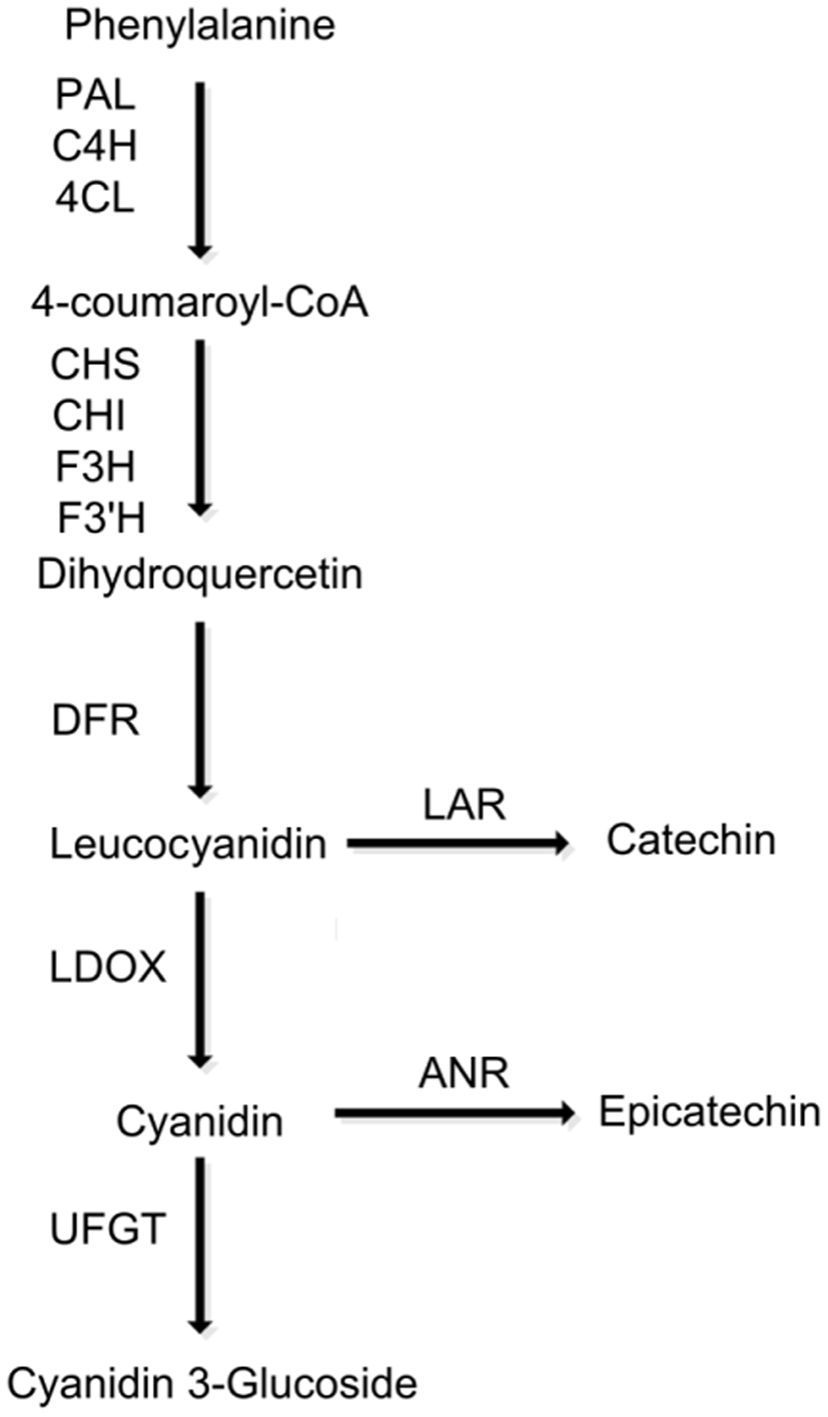
**Schematic diagram of the flavonoid biosynthetic pathway in peach.** PAL, phenylalanine ammonia-lyase; C4H, cinnamate 4-hydroxylase; 4CL, 4-coumarate:CoA ligase; CHS, chalcone synthase; CHI, chalcone isomerase; F3H, flavanone 3-hydroxylase; F3′H, flavonoid 3′-hydroxylase; DFR, dihydroflavonol reductase; LODX, leucoanthocyanidin dioxygenase; UFGT, UDP glucose, flavonoid 3-*O*-glucosyltransferase.

The biosynthesis of PAs shares common steps with the anthocyanin pathway until the flavan-3,4-diol step (e.g., leucocyanidin in **Figure 1**). Leucocyanidin is converted into flavan-3-ols catechin and epicatechin through either via a single-step reaction catalyzed by leucoanthocyanidin reductase (LAR) or a two-step reaction catalyzed by leucoanthocyanidin dioxygenase (LDOX) and anthocyanidin reductase (ANR), respectively. However, little is known about the polymerization of flavan-3-ol monomers. To date, PA-specific pathway genes, *LAR* and *ANR*, have been characterized in a variety of fruit trees, such as grapevine (Bogs et al., 2005), persimmon (Ikegami et al., 2005, 2007; Akagi et al., 2009a,b), apple (Han et al., 2012; Henry-Kirk et al., 2012; Liao et al., 2015), strawberry (Schaart et al., 2013; Fischer et al., 2014), and peach (Ravaglia et al., 2013).

Proanthocyanidin accumulation is regulated at the transcriptional level by MYB transcription factors (TFs). To date, PA-related MYB activators have been identified in various plant species, such as *AtMYB123* (*AtTT2*) and *AtMYB5* in *Arabidopsis* (Nesi et al., 2001; Li et al., 2009), *VvMYBPA1, VvMYBPA2, VvMYB5a*, and *VvMYB5b* in grapevine (Deluc et al., 2006, 2008; Bogs et al., 2007; Terrier et al., 2009), *MtPAR, MtMYB5*, and *MtMYB14* in *Medicago* (Verdier et al., 2012; Liu et al., 2014), *LjTT2s* in lotus (Yoshida et al., 2008), *DkMYB2* and *DkMYB4* in persimmon (Akagi et al., 2009b, 2010), *PtMYB134* in poplar (Mellway et al., 2009), *TaMYB14* from *Trifolium arvense* (Hancock et al., 2012), *FtMYB1*/*FtMYB2* in tartary buckwheat (Bai et al., 2014), *PpMYBPA1* in nectarine (Ravaglia et al., 2013) and *MdMYB9*/*MdMYB11* in apple (Gesell et al., 2014; An et al., 2015). These PA-related MYB activators are divided into three phylogenetic groups (Akagi et al., 2010; Hancock et al., 2012).

Proanthocyanidin biosynthesis is affected by internal developmental cues such as phytohormones and external environmental stresses such as wounding (Zoratti et al., 2014; Liu et al., 2015). In fruit trees, the effects of developmental and environmental cues on PA synthesis have been investigated in persimmon as its fruit accumulates abundant PAs. DkbZIP5 can activate the PA-related MYB activator DkMYB4 via an ABA signal, resulting in the accumulation of PAs (Akagi et al., 2012). Whereas, ethylene responsive factors (ERFs) induce a decrease of PAs via the ethylene pathway (Min et al., 2012). Wounding induces PA synthesis through activating PA-related MYB regulator *DkMYB2* (Akagi et al., 2010). More recently, jasmonate has been shown to activate PA synthesis in apple (An et al., 2015).

In *Arabidopsis*, there are 125 R2R3-MYB TFs in the genome (Stracke et al., 2001), with many yet to be functionally characterized. In this work, we isolated and characterized an R2R3-MYB TF, designated *PpMYB7*, in peach. Phylogenetic analysis indicates that the *PpMYB7* gene and *MYB* genes from other species form an outgroup of previously reported PA-MYB activators that affect transcription of both *LAR* and *ANR* genes. However, PpMYB7, unlike PpMYBPA1, only activates transcription of *PpLAR1*, but not for *PpANR*. Our study aids in the understanding of the functional divergence between R2R3-MYB TFs in plants, and in manipulating PA synthesis in peach.

## Materials and Methods

### Plant Materials

The variety ‘Baifeng’ of Peach (*Prunus persica*) is maintained at Wuhan Botanical Garden of the Chinese Academy of Sciences (Wuhan, Hubei Province, China). Fruit samples were collected at 30 (fruitlet), 65 (pre-ripening), 85 (early ripening stage), and 95 (full ripening stage) DAFB (days after full bloom) in 2012 and each stage consisted of 10 fruits. Fruits were mechanically peeled and cored, and the flesh was cut into small sections. Fruit samples for each stage were mixed and immediately frozen in liquid nitrogen, and then stored at -75°C until use.

### Construction of Expression Vectors

A pair of primers, MYB7topoF and MYB7topoR, was designed to amplify the coding sequences of *PpMYB7* using iProof High-Fidelity PCR kit (Bio-Rad, Hercules, CA, USA) and cDNA templates from fruit tissues of peach ‘Baifeng.’ PCR products were purified and inserted into pENTR/D-TOPO (Invitrogen, Carlsbad, CA, USA) according to the manufacturer’s instructions. The positive clone was validated by direct sequencing and then inserted into binary vector pHEX2 using LR recombination cloning according to the manufacturer’s instructions (Invitrogen, Carlsbad, CA, USA). Similarly, the coding sequences of *PpbHLH3* and *PpbHLH33* were also cloned and inserted individually into pHEX2 vector using the same protocol as described for the *PpMYB7* gene. In addition, a pair of primers, PpMYBPA1OEF (with a tail containing a *Hind*III site at the 5′ end) and PpMYBPA1OER (with a tail containing an *Xba*I site at the 5′ end), was designed to amplify the full coding region of *PpMYBPA1*. PCR products were digested with *Hind*III and *Xba*I and then inserted into pSAK277 vector. All the sequences of primers used for expression vector construction are listed in Supplementary Table S1.

### Yeast Two Hybrid Assay

Yeast two-hybrid analysis was performed using Matchmaker Gold Yeast One-Hybrid Library Screening System (Clontech, Palo Alto, CA, USA). Firstly, the autoactivation activities of PpMYB7, PpbHLH3, and PpbHLH33 were tested. The full coding regions of the three genes were inserted into pGBKT7 vector. The primer sequences are listed in Supplementary Table S1. Empty pGBKT7 vector and the above three constructs were transformed individually into yeast strain Y2Hgold. The positive colonies were grown on SD-Trp and SD-Trp-Ade-His medium, and the results were observed after 3 days. The full coding region of *PpMYB7* was inserted into pGADT7 vector, while partial coding sequences of *PpbHLH3* (amino acid 1–235) and *PpbHLH33* (amino acid 1–235) were inserted individually into pGBKT7 vector. The primer sequences are listed in Supplementary Table S1. Empty vector pGADT7 and its derivations were transformed individually into yeast strain Y187, whereas, empty vector pGBKT7 and its derivations were transformed individually into yeast strain Y2Hgold. After matting in 2 × YPDA medium, the positive colonies were grown on different types of medium, respectively, including DDO (SD-Trp-Leu), QDO/A (SD-Trp-Leu-Ade-His+AbA), and QDO/X/A (SD-Trp-Leu-Ade-His+X-α-Gal+AbA). Photographs were taken 3 days after incubation.

### Dual Luciferase Reporter Assay

A dual luciferase reporter assay was conducted in *Nicotiana benthamiana* leaves according to a previous report (Espley et al., 2007). Upstream regions from the ATG start site of four peach genes, *DFR* (1.6 Kb), *LAR1* (1.4 Kb), *ANR* (1.9 Kb), and *UFGT* (2.5 Kb), were isolated and inserted into multiple cloning site of vector pGreen 0800-LUC (Hellens et al., 2005). All the constructs were transformed into *Agrobacterium tumefaciens* GV3101, and incubated at 28°C for 2 days. The confluent bacteria was resuspended in infiltration buffer (10 mM MgCl_2_, 0.5 μM acetosyringone) and incubated at room temperature without shaking for 2 h before infiltration. Transient transformation was conducted by mixing 100 mL *Agrobacterium* strain GV3101 culture transformed with the reporter cassette with 450 μL *Agrobacterium* culture transformed with a cassette containing *PpMYBPA1, PpMYB7, PpbHLH3*, or *PpbHLH33* fused to the 35S promoter. All analyses were repeated at least four times using biological replicates. The ratio of LUC to Ren activity was measured 3 days after infiltration using Dual-Glo^®^ Luciferase Assay System (Promega Corporation, Madison, WI, USA).

### Firefly Luciferase Complementation Assay

Firefly luciferase complementation assay was conducted using *N. benthamiana* young leaves according to a previous report (Chen et al., 2008). The full coding region of *PpMYB7* was inserted into binary vector pCambia1300-NLuc, while the full coding regions of PpbHLH3 and PpbHLH33 were inserted individually into pCambia1300-CLuc. The primer sequences are listed in Supplementary Table S1. Preparation and infiltration of *A. tumefaciens* were performed using the same protocol as described for dual luciferase reporter assay. Luminescence units were measured using an Infinite M200 luminometer (Tecan, Mannerdorf, Switzerland). Leaf disks (exactly 2 cm in diameter) were punched adjacent to the infiltration site and firefly luciferase activity was assayed using Steady-Glo^®^ Luciferase Assay System^®^ (Promega Corporation, Madison, WI, USA) according to the manufacturer’s instructions.

### Quantification of Flavan-3-ols in Peach Fruits

Peach fruit sample was ground into fine powder, and 10 mg of powder was added to 1 mL of 70% (v/v) acetone containing 0.1% (w/v) ascorbic acid. The mixture was incubated at room temperature for 24 h in darkness. The extract was centrifuged and the supernatant was transferred to a 1.5 ml microcentrifuge tube. The extract was purified by adding equal amount of chloroform and the supernatant was collected. The solvent was evaporated, and the extract was resuspended in 500 μL of water/methanol (1:1, v/v). PAs were quantified using high-performance liquid chromatography (HPLC) according to our previously reported protocol (Liao et al., 2015). Briefly, the HPLC Separation was performed on a Hisep C18-T column (5 μm, 4.6 mm × 150 mm; Weltech, Co., Ltd., Wuhan, China). The PAs were observed under UV detector at 280 nm and determined according to retention time of standards, including catechin and epicatechin (Sigma). All analyses were repeated three times using biological replicates.

### Quantitative Real-Time PCR (qRT-PCR)

Total RNA was extracted using Total RNA Rapid Extraction Kit (Zomanbio, Beijing, China). First strand cDNA was synthesized using PrimeScript^@^ Reverse Transcriptase (TaKaRa, Dalian, China). qRT-PCR was performed in a total volume of 20 μL reaction containing 100 ng of template cDNA, 0.2 μM of each primer, and 10 μL of 2 [SYBR premix Ex TaqTM (TaKaRa)]. The amplification program was as follows: one cycle of 30 s at 95°C, followed by 40 cycles of 5 s at 95°C, 34 s at 60°C. PpTEF2 (GDR accession no. ppa001368m) was selected as an internal control according to a previous report (Tong et al., 2009). The standard curve method was conducted to quantify the transcripts. All analyses were repeated three times. The sequences of primers used for real-time PCR analysis are listed in Supplementary Table S2.

## Results

### Identification of an R2R3-MYB Gene, *PpMYB7*, Which Potentially Regulates PA Synthesis in Peach

We have previously generated an RNA-Seq-based transcriptome database of peach (Wang et al., 2013). Screening the RNA-Seq data revealed two MYB TFs which showed high level of expression in fruits prior to ripening. One is *PpMYBPA1*, which is implicated in the regulation of PA accumulation in nectarine (Ravaglia et al., 2013). Another, designated *PpMYB7* (NCBI accession no. KT159231), is a typical R2R3-MYB. The coding sequence of *PpMYB7* in cv. Baifeng is identical to that of a predicted gene in cv. Lovell deposited in Genome Database for Rosaceae (GDR^1^) with an accession no. ppa016135m. It encodes a protein of 203 amino acid residues that is relatively shorter than most of R2R3-MYB proteins. The closest homolog of PpMYB7 is an MYB protein (GenBank accession no. XP_008238440) from *Prunus mume* of unknown function. PpMYB7 shows a low level (<40%) of identity in amino acid sequence with MYBs involved in the regulation of anthocyanins or PAs, such as AtPAP1 and AtMYB123 in *Arabidopsis* and VvMYBPA1 and VvMYBPA2 in grapevine.

**FIGURE 2:**
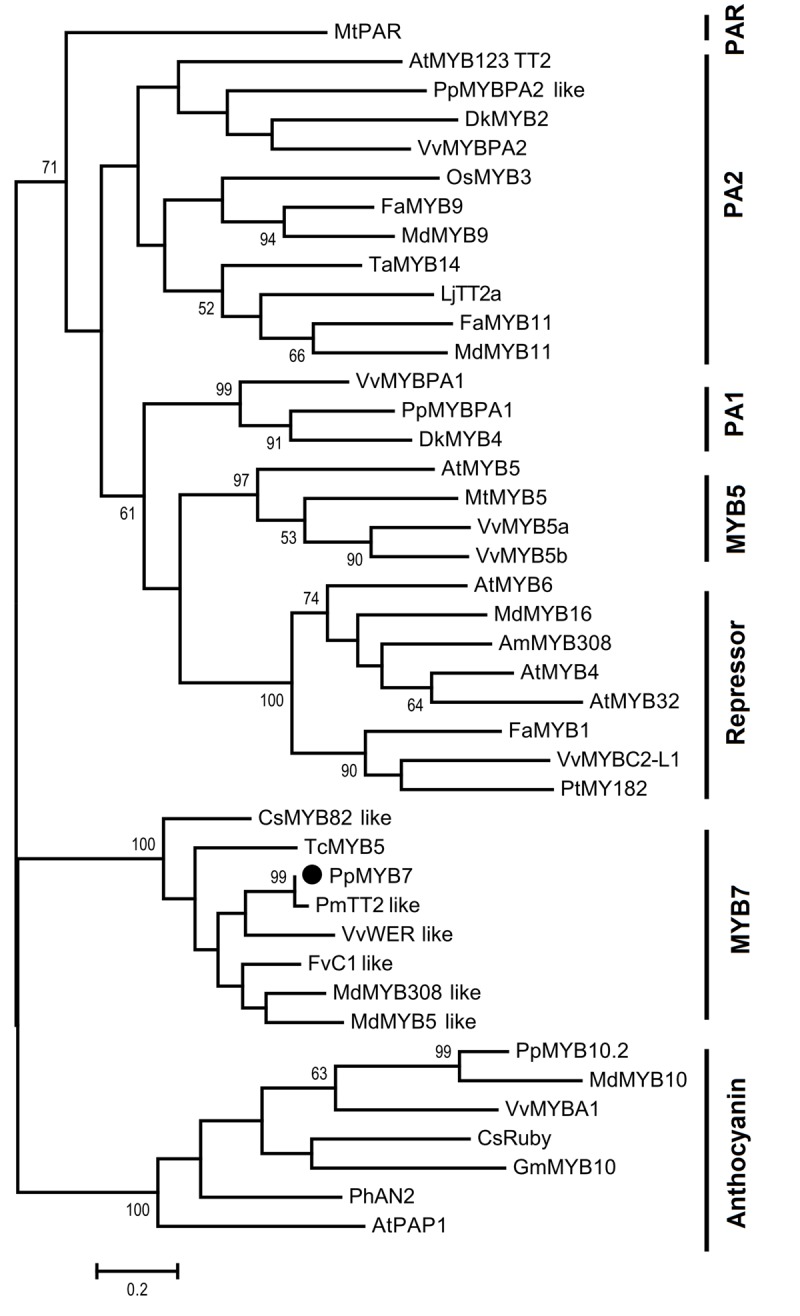
**Phylogenetic tree derived from amino acid sequences of genes encoding R2R3-MYB transcription factors.** The *PpMYB7* gene isolated in this study is highlighted with a black circle. The full length amino acid sequences were aligned using Muscle software and phylogenetic tree was conducted with MEGA version 6.0 using the maximum likelihood method. Numbers on branches represent bootstrap estimates for 1,000 replicate analyses and values <50% are not indicated. The scale bar represents 0.2 substitutions per site. The GenBank or TAIR accession numbers are as follows: *Arabidopsis thaliana* AtMYB4 (AT4G38620), AtMYB5 (AT3G13540), AtMYB6 (AT4G09460), AtPAP1 (AT1G56650), AtMYB123 (AT5G35550), AtMYB32 (AT4G34990), *Vitis vinifera* VvMYBA1 (AB097923), VvMYBPA2 (EU919682), VvMYBPA1 (AM259485), VvMYB5a (AAS68190), VvMYB5b (AAX51291), VvMYBC2-L1 (JX050227), VvWER_like (XP_010646852); *Prunus persica* PpMYB10.2 (EU155160), PpMYB7 (KT159231), PpMYBPA1 (CV047374), and PpMYBPA2 like (XM_007203070); *Malus domestica* MdMYB308_like (XP_008369485), MdMYB5_like (XP_008356551), MdMYB10 (DQ267897), MdMYB16 (HM122617), MdMYB9 (ABB84757), MdMYB11 (AAZ20431); *Fragaria x ananassa* FaMYB9 (JQ989281), FaMYB11 (JQ989282), FaMYB1 (AF401220); *Oryza sativa* OsMYB3 (D88619); *Diospyros kaki* DkMYB2 (AB503699), DkMYB4 (AB503701); *Trifolium arvense* TaMYB14 (JN049641); *Petunia x hybrid* PhAN2 (ABO21074); *Citrus sinensis* CsRuby (NM_001288889), CsMYB82_like (XP_006477150); *Garcinia mangostana* GmMYB10 (FJ197137); *Lotus japonicas* LjTT2a (AB300033); *Fagopyrum tataricum* FtMYB1 (AEC32973); *Antirrhinum majus* AmMYB308 (P81393); *Prunus mume* PmTT2_like (XP_008238440); *Fragaria vesca* FvC1_like (XP_004299414); *Theobroma cacao* TcMYB5 (XP_007039783); *Populus tremula x Populus tremuloides* PtMYB182 (KP723392), *Medicago truncatula* MtMYB5 (XP_003601609), MtPAR (HQ337434).

**FIGURE 3:**
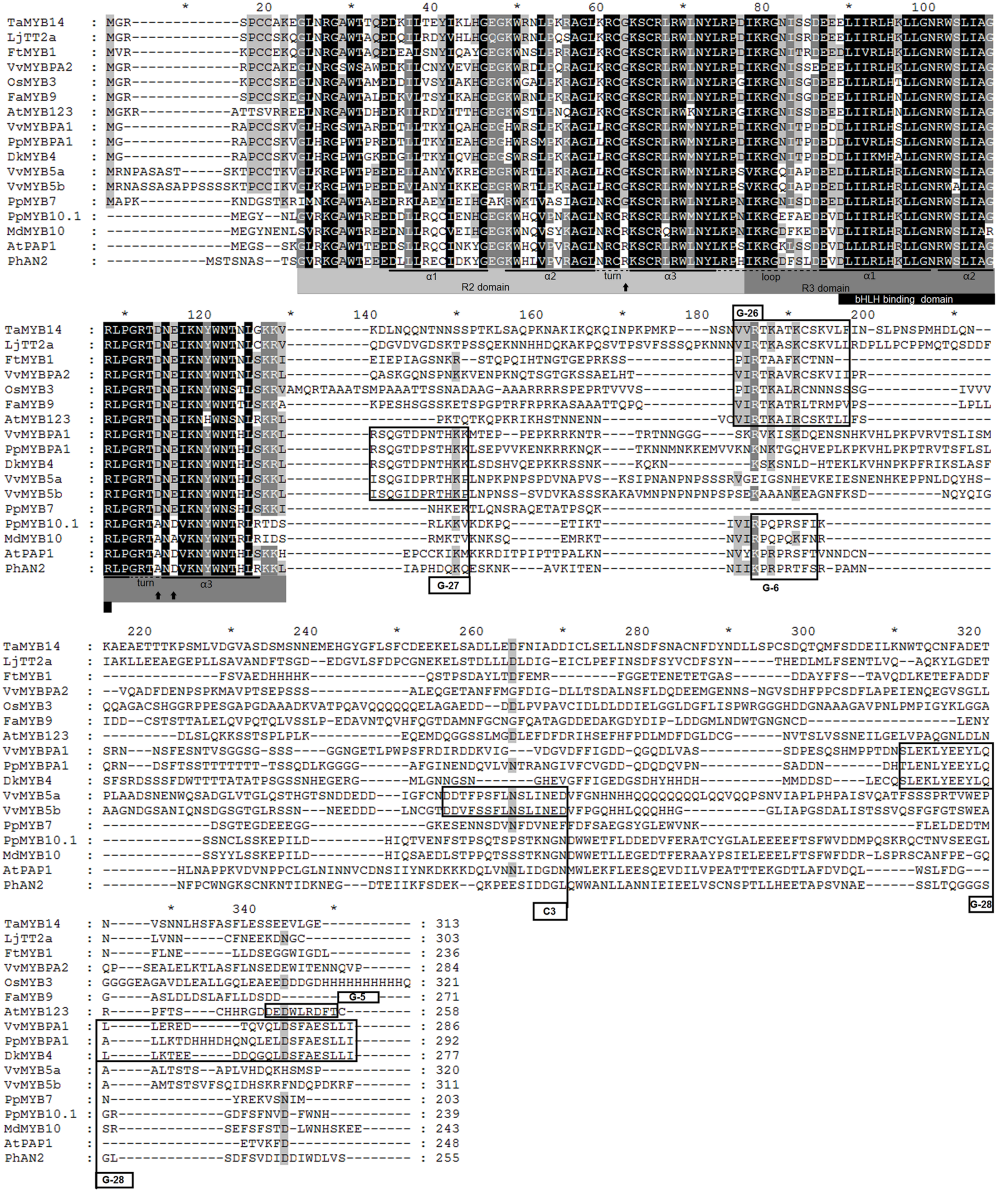
**Amino acid sequence alignment of the full length amino acid sequences of PpMYB7 and other known proanthocyanidin and anthocyanin MYB regulators in plants.** Conserved residues are highlighted in black, and partial conservation is indicated in gray. The alpha helices of R2 and R3 repeats are indicated with black lines. Black arrows indicate key residues that mediate specificity for either the anthocyanin or PA pathway (Heppel et al., 2013). Conserved motifs in the C-terminal region (G-5, G-6, G-26, G-27, and G-28) were numbered according to Stracke et al. (2001) and Heppel et al. (2013), and C3 motif according to Deluc et al. (2008).

Initially, we compared the *PpMYB7* gene with all R2R3 MYB TFs in *Arabidopsis* and found it was closely related to *TT2/AtMYB123* (Supplementary Figure S1). Subsequently, phylogenetic analysis was further conducted for PpMYB7 and MYB TFs in various plants. Surprisingly, the result showed that *PpMYB7* does not fall into any groups of *MYB* genes with known function for flavonoid synthesis, but belongs to a new group (designated MYB7) with unknown function (**Figure 2**). Amino acid sequence alignment indicated that previously reported flavonoid-related MYB TFs contain one or more motifs in C-terminal region (Stracke et al., 2001), but PpMYB7 does not contain any motifs in C-terminal region (**Figure 3**). This result further confirms that *PpMYB7* is different from previously reported flavonoid-related MYB TFs. In addition, a residue at positions 39 in the R2 domain and four residues at positions 90–93 in the R3 domain are key amino acid elements that control the specificity of MYB regulators for either the anthocyanin or PA pathway (Heppel et al., 2013). The key amino acid elements in PpMYB7 are the same as those in MYBs related to PAs but not anthocyanins (**Figure 3**), which suggests PpMYB7 is likely involved in the regulation of PA accumulation in peach.

### Expression Profiles of *MYB* Regulators and Structural Genes of PA Synthesis in Peach Fruits

We screened our RNA-Seq database of peach fruit transcriptome (Wang et al., 2013) and three candidate genes encoding biosynthetic steps of PA synthesis were identified, including *PpANR* gene (ppa008295m), *PpLAR1* (ppa009439m), and *PpLAR2* (ppa016135m). Expression profiles of these three genes and two *MYB* regulators, *PpMYB7* and *PpMYBPA1*, were investigated in peach fruits using qRT-PCR analysis and two biological replicates was conducted in two successive years 2012 and 2013. Since the result in 2013 (Supplementary Figure S2) is similar to that in 2012, only the result in 2012 was described here.

**FIGURE 4:**
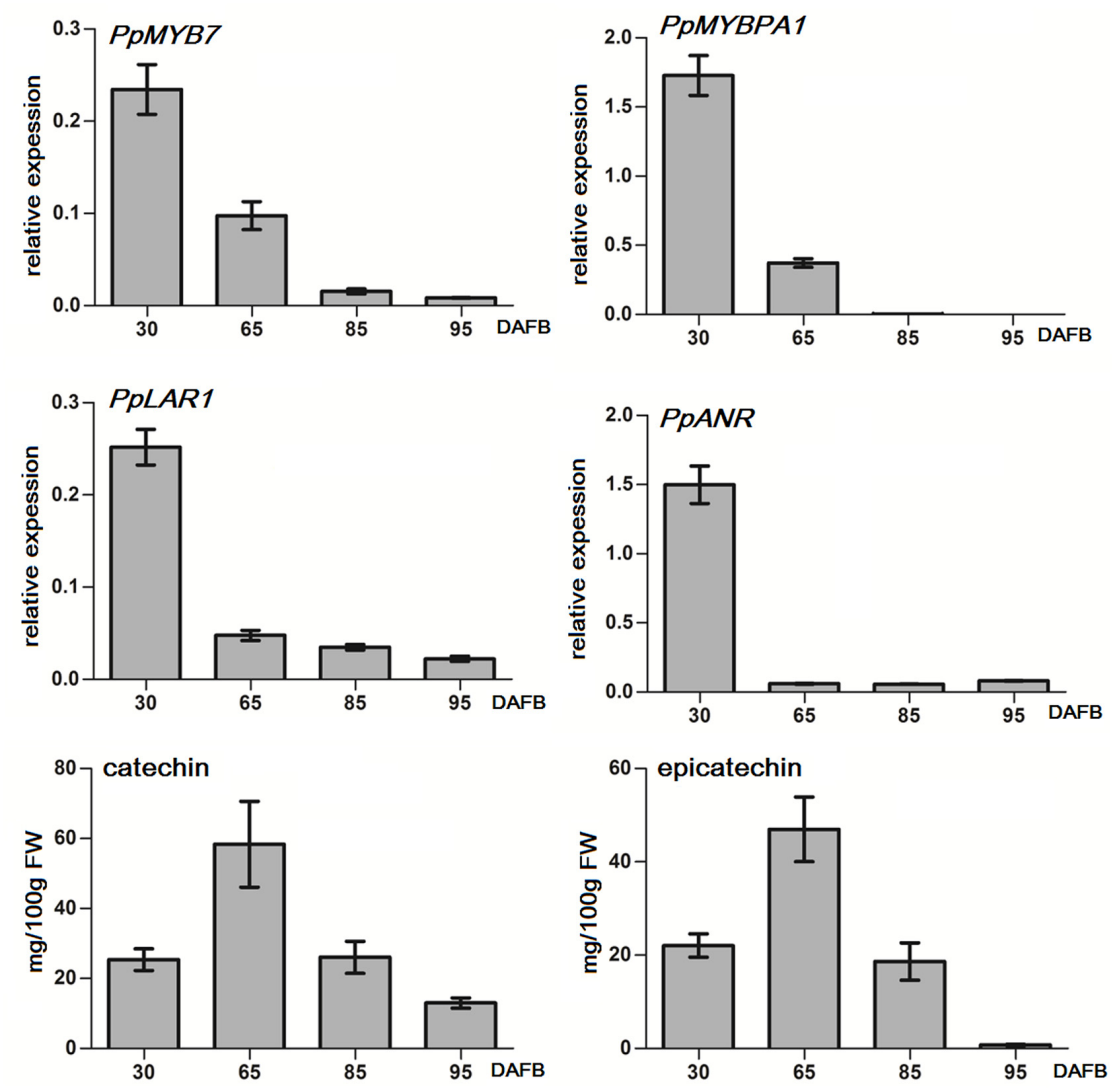
**Quantitative real-time PCR (qRT-PCR) analysis of the expression profiles of genes related to PA synthesis and flavan-3-ol content in fruits of peach cv.** Baifeng that were at different stages of development in 2012. qRT-PCR was done for three technical replicates. Catechin and epicatechin contents represent the means of three biological repeats. Error bars show SE of the mean. DAFB stands for days after full bloom. A biological replicate was conducted for fruit samples collected in 2013, and the results (Supplementary Figure S2) are similar to those in 2012.

Both *PpMYB7* and *PpMYBPA1* showed a decreasing expression trend throughout fruit development, and their transcript levels were extremely low or almost undetectable after 85 DAFB (days after full bloom; **Figure 4**). Similarly, both *PpLAR1* and *PpANR* had the highest level of expression in fruits at 30 DAFB, and showed a decrease in expression throughout fruit development. The transcript levels of *PpLAR1* and *PpANR* decreased significantly after 65 DAFB. In addition, the transcript of *PpLAR2* was undetectable at all tested stages of fruit development (data not shown). Therefore, the expression level of *PpMYB7* and *PpMYBPA1* is correlated with that of *PpLAR1* and *PpANR* in peach fruits.

In addition, we also investigated the accumulation of flavan-3-ols in flesh throughout fruit development (**Figure 4**). The accumulation of both catechin and epicatechin showed an increase during early fruit developmental stages, with a peak at 65 DAFB at the S3 development stage, and then decreased during ripening. The accumulation of epicatechin was almost undetectable at the full ripening stage (95 DAFB). These results suggest that the expression of *MYB* regulators and the genes encoding biosynthetic steps of PA synthesis precedes the accumulation of flavan-3-ols in peach fruits.

### PpMYB7 Specifically Activates *PpDFR* and *PpLAR1*

**FIGURE 5:**
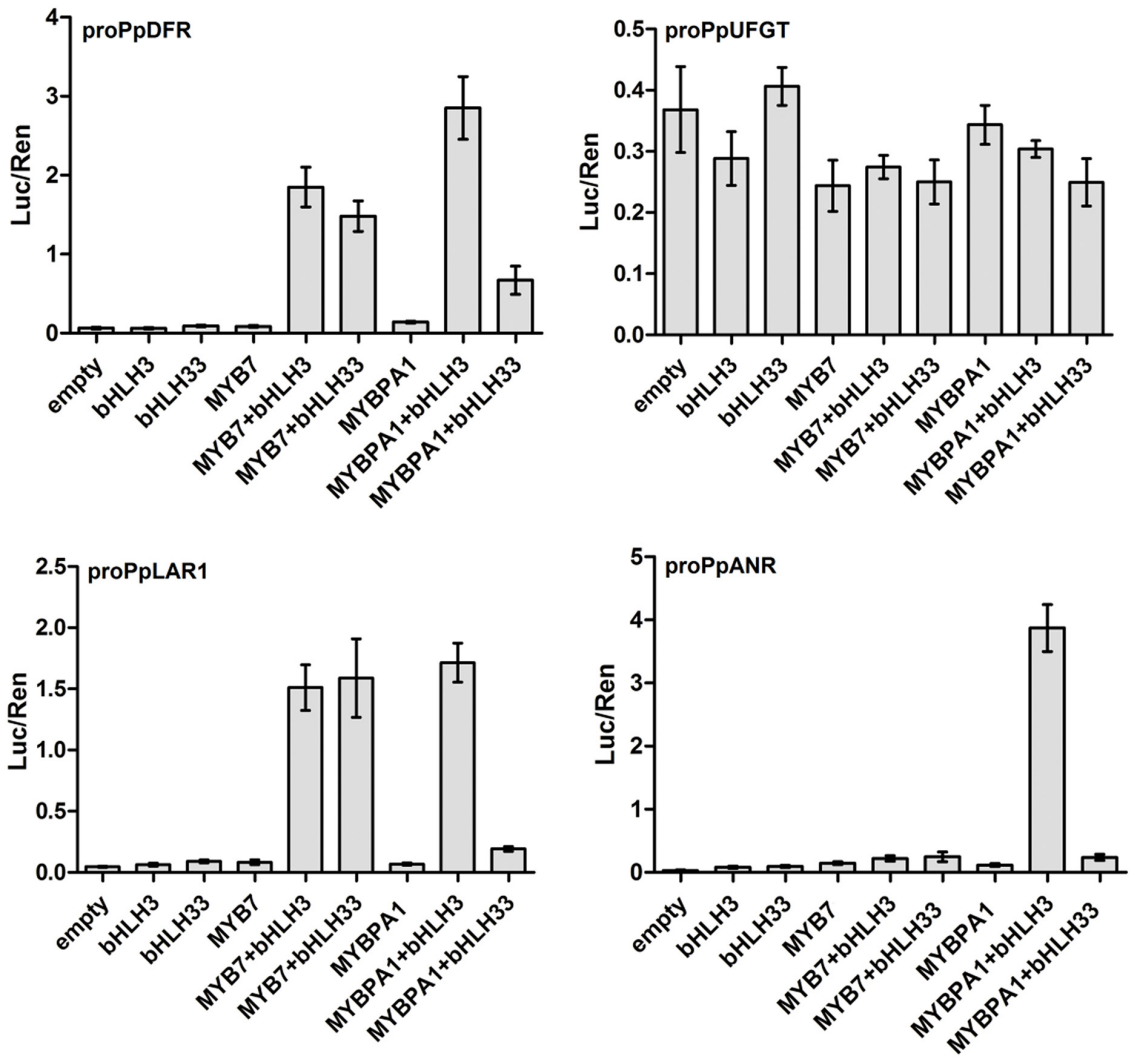
**Analysis of the interaction of PpMYB7 and PpMYBPA1 with the promoters of *PpDFR, PpLDOX, PpLAR1*, and *PpANR* in transiently transfected *Nicotiana benthamiana* leaves.** Luc and Ren were measured 3 days after infiltration, and error bars show SE of four biological replicates.

To elucidate the role of PpMYB7 in the regulation of PA accumulation, dual luciferase reporter assays of promoter activity were conducted. As both peach MYB regulators had residues that predicted interaction with bHLH TFs to activate the flavonoid pathway genes (Zimmermann et al., 2004; Hichri et al., 2011), two peach bHLHs (PpbHLH3 and PpbHLH33) that are expressed in fruits (Zhou et al., 2015) were selected as candidate partners of PpMYB7. PpbHLH3 belongs to bHLH2/AN1/TT8 clade and PpbHLH33 belongs to bHLH1/JAF13/EGL3 clade. Our previous study showed that PpbHLH3 rather than PpbHLH33 participates in regulation of anthocyanin synthesis in fruits (Zhou et al., 2015). Four genes of the phenylpropanoid biosynthetic pathway, *PpDFR, PpLAR1, PpANR*, and *PpUFGT*, were selected to test the interaction of their promoters with PpMYB7. Infiltration of PpMYB7, bHLH3, or bHLH33 alone resulted in very low activity against the promoters of all the tested genes (**Figure 5**). Co-infiltration of PpMYB7 with PpbHLH3 or PpbHLH33 showed a significant increase in *PpDFR* and *PpLAR1* promoter activity, with ratios of LUC to REN ranging from 1.6 to 2.0. In contrast, co-infiltration of PpMYB7 with PpbHLH3 or PpbHLH33 showed little activity against both *PpUFGT* and *PpANR* promoters. These results suggest that the *PpMYB7* gene regulates the biosynthesis of catechin but not epicatechin in peach.

Unlike PpMYB7, PpMYBPA1 had good activity against both *PpLAR1* and *PpANR* promoters and this activity required PpbHLH3 but not PpbHLH33. Similarly, a high activity was observed for the *PpDFR* promoter when PpMYBPA1 was co-transformed with PpbHLH3, whereas, co-transformation of PpMYBPA1 and PpbHLH33 failed to activate the promoter as much as that of PpMYBPA1 and PpbHLH3. In addition, MYBPA1, like PpMYB7, also showed very low activity against the *PpUFGT* promoter.

### PpMYB7 Binding to PpbHLH3 and PpbHLH33 *In vivo*

**FIGURE 6:**
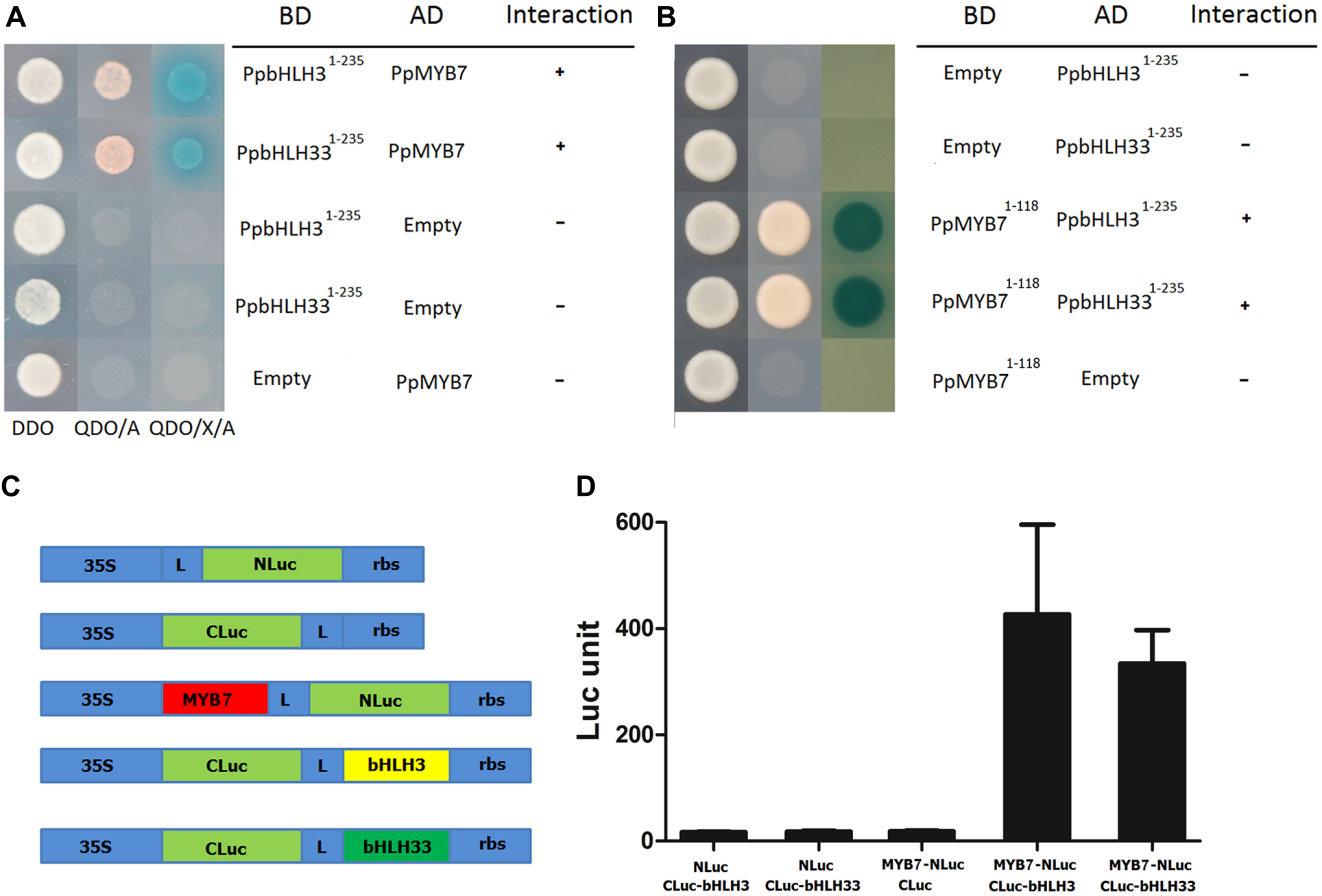
**Interaction of PpMYB7 with PpbHLH3 or PpbHLH33 *in vivo*. (A,B)** Yeast two hybrid assay. DDO, SD-Trp-Leu medium; QDO/A, SD-Trp-Leu-Ade-His+AbA medium; QDO/X/A, SD-Trp-Leu-Ade-His+X-α-Gal+AbA medium. **(C)** Schematic diagram of the NLuc, CLuc, and NLuc/CLuc constructs. L, Gly/Ser linker; rbs, Transcription terminator derived from the Rubisco small subunit gene. **(D)** Firefly luciferase complementation assay in young *N. Benthamiana* leaves. The error bars stand for SE of six biological replicates.

Interaction of PpMYB7 with PpbHLH3 and PpbHLH33, which was observed in dual luciferase reporter assay as described above, was further tested using both yeast two-hybrid (Y2H) and firefly luciferase complementation assays. Firstly, the autoactivation was tested for PpMYB7, PpbHLH3, and PpbHLH33. The full coding regions of the three TFs were amplified and inserted into pGBKT7 vector. Autoactivation test results showed that PpMYB7-pGBKT7 and PpbHLH33-pGBKT7 were able to grow in SD-Trp-Ade-His medium, but not for the empty pGBKT7 or PpbHLH3-pGBKT7 (Supplementary Figure S3). This indicates that PpMYB7 and PpbHH33 have autoactivation ability. To avoid any false positive results and to keep the consistency of the two bHLHs, both PpbHLH3 and PpbHLH33 were truncated and their N-terminal domain (amino acid residues 1–235) was amplified and subjected to Y2H assay according to previously reported method (Hernandez et al., 2004). Empty-AD and empty-BD vectors were used as negative controls. Transformants with the PpMYB7 and the N-terminal domain of PpbHLH3 or PpbHLH33 showed growth on both DDO and QDO/A media, and their color turned to blue when grown on QDO/X/A medium (**Figure 6A**). In contrast, the negative controls could grow on DDO medium, but not on QDO/A medium. Moreover, R2R3 domain of PpMYB7 (amino acid 1–118) was also inserted into pGBKT7 vector to test its interaction with PpbHLH^1-235^ and PpbHLH33^1-235^, and the result showed that PpMYB7^1-118^ was able to interact with both PpbHLH^1-235^ and PpbHLH33^1-235^ (**Figure 6B**).

In addition, PpMYB7 was fused to N-terminus of luciferase (NLuc), while PpbHLH3 or PpbHLH33 was fused to C-terminus of luciferase (CLuc; **Figure 6C**). Empty-NLuc and empty-CLuc vectors were used as controls. Co-expression of PpMYB7-NLuc and bHLH3-CLuc or bHLH33-CLuc was able to rescue intense luciferase activity, which did not occur in any controls, bHLH3 or bHLH33-CLuc with NLuc and PpMYB7-NLuc with CLuc (**Figure 6D**). Taken together, these results indicate that PpMYB7 can bind to both PpbHLH3 and PpbHLH33 *in vivo*.

### *Cis*-regulatory Elements in the Promoter Region of *PpMYB7* and *PpMYBPA1*

**FIGURE 7:**
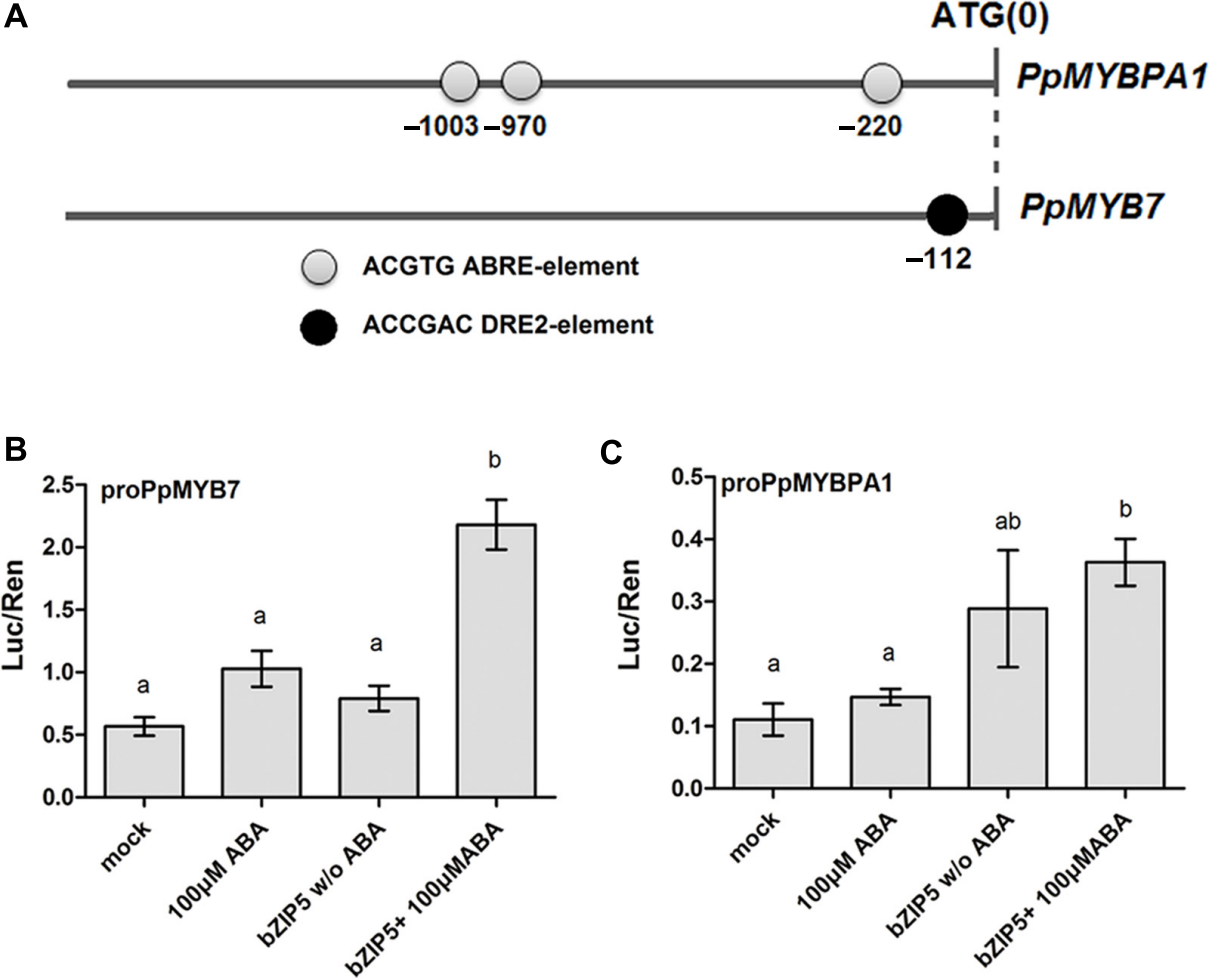
**Functional analysis of the promoter sequences of PpMYB7 and PpMYBPA1. (A)** *Cis*-regulatory elements in the promoter sequences of *PpMYBPA1* and *PpMYB7*. **(B,C)** Analysis of the interaction of PpbZIP5 and ABA with the promoters of *PpMYB7* and *PpMYBPA1* in transiently transfected *N. benthamiana* leaves. Mock represents the control soaked in sterile distilled water. Different lowercase letters indicate the significant differences among treatments by Tukey’s test at *P* < 0.05. Error bars show SE of the mean (four biological replicates).

A 1.9-kb fragment upstream of the ATG start codon of *PpMYB7* and *PpMYBPA1* was examined to identify *cis*-regulatory elements using the PLACE software^2^. As a result, three abscisic acid response elements (ABREs) were found at positions -220, -970, and -1003 bp upstream of the start codon of *PpMYBPA1*, whereas, a drought-responsive element 2 (DRE2) was found at position -112 from the start codon of *PpMYB7* (**Figure 7A**). ABA-dependent DkbZIP5 can recognize ABREs to induce the expression of PA-specific regulator *DkMYB4* in persimmon (Akagi et al., 2012). The coding sequence of the *DkbZIP5* gene was compared against the peach reference genome (Verde et al., 2013), and eight homologs were identified. Phylogenetic analysis indicated that an ABF-, AREB-, and ABI5-Like bZIP showed the closest relationship to *DkbZIP5* (Supplementary Figure S4) and was designated *PpbZIP5* (NCBI accession no. KT223015). The coding sequence of *PpbZIP5* was isolated to conduct a dual luciferase assay. As expected, PpbZIP5 was able to activate the promoters of both *PpMYB7* and *PpMYBPA1* in the presence of exogenously supplied ABA (**Figures 7B,C**).

## Discussion

### Peach *PpMYB7* Represents a New Group of R2R3-MYB Genes Regulating PA Synthesis in Plants

In plants, PA biosynthesis is regulated by the MBW complex formed by interaction of three types of TFs: R2R3-MYBs, basic helix-loop-helix (bHLH) proteins and WD40 repeats. In this complex, MYB TFs play a critical role in recognizing *cis*-elements in the promoters of PA pathway genes (Schaart et al., 2013). Here, phylogenetic analysis reveals that the previously reported PA-related R2R3-MYB activators are divided into four groups (**Figure 2**). Three groups, PA1 (Bogs et al., 2007; Akagi et al., 2009b), PA2 (Baudry et al., 2004; Terrier et al., 2009; Hancock et al., 2012; Liu et al., 2014), and MYB5 (Deluc et al., 2008; Xu et al., 2014), contain members from various plant species, while one group PAR only contains one member *MtPAR* from *Medicago truncatula* (Verdier et al., 2012). In addition, one group which is characterized as repressors closely clusters with PA1 and MYB5 groups (Cavallini et al., 2015). It is worth noting that some *MYB* repressors, such as *FaMYB1* (Aharoni et al., 2001), *PtMYB182* (Yoshida et al., 2015), and *VvMYBC2-L1* (Huang et al., 2014) are related to the biosynthesis of anthocyanins and PAs.

Our study reveals functionally a new MYB7 group, which consists of the peach *PpMYB7* and its homologs in species as diverse as citrus, grape, strawberry, and apple (**Figure 2**). The *MYB* genes within this group, like the PA2 group MYBs, consist of three exons and two introns, and are more closely related to the PA-related MYBs than to the anthocyanin-related MYBs. Dual luciferase assays demonstrate that PpMYB7 can activate transcription of PA-specific pathway gene *PpLAR*, but not for the anthocyanin-specific gene UFGT *PpUFGT*. To date, all the characterized anthocyanin-related MYBs, such as *AtMYB75*/*AtPAP1* (Borevitz et al., 2000), *VvMYBA1* (Walker et al., 2007), *MdMYB10* (Espley et al., 2007), and *PpMYB10.1* (Zhou et al., 2015), are able to activate transcription of *UFGT*. Thus, the dual luciferase assay clearly suggests that the *PpMYB7* gene is a PA-related MYB regulator.

Nevertheless, the *PpMYB7* gene is functionally different from the *PpMYBPA1* gene, a member in the PA1 group. The *PpMYB7* gene activates transcription of *PpLAR* but not *PpANR*, whereas, the *PpMYBPA1* gene can activate transcription of both *PpLAR* and *PpANR*. Similar to *PpMYBPA1*, all the previously reported PA-related MYB activators except *VvMYB5a* can also induce the transcription of both *PpLAR* and *PpANR*. The *VvMYB5a* gene shares a similar activation pattern with the *PpMYB7* gene although they do not have an orthologous relationship. In grapevine, the *VvMYB5a* gene can only activate *VvLAR*, but its ectopic expression also activates transcription of *AtBAN* encoding ANR in *Arabidopsis* and induces the accumulation of epicatechin but not catechin in tobacco (Deluc et al., 2006, 2008). It is known that the α-helices of the R2R3 domain play a critical role in directing MYBs to bind the promoters of flavonoid structural genes (Ogata et al., 1994; Williams and Grotewold, 1997; Jia et al., 2004). The third α-helix of the R2 domain and all the three α-helices of the R3 domain are conserved between PpMYB7 and the PA-related MYB activators, but they show divergence in the first and second α-helices of the R2 domain. The first two flexible α-helices of the R2 domain are likely important in the recognition of a specific gene target as they contribute to secondary structure and ternary folding of the R2R3 domain (Jia et al., 2004). More research is needed to address if the divergence in activation pattern between PpMYB7 and other PA-related MYB activators has arisen from their genetic variation in the first and second α-helices of the R2 domain.

Proanthocyanidin-related MYBs require a bHLH partner for the *trans*-activation of PA pathway genes (Bogs et al., 2007; Deluc et al., 2008). For example, MdMYB9 requires MdbHLH3 as a partner to regulate the biosynthesis of PAs in apple fruit (Gesell et al., 2014; An et al., 2015). bHLH TFs involved in the regulation of the flavonoid pathway from a variety of species are divided into two clades, bHLH2/AN1/TT8 and bHLH1/JAF13/EGL3 (Montefiori et al., 2015).

A previous study indicates that PpMYBPA1 requires a partner PpbHLH3 to activate transcription of *PpLAR* (Ravaglia et al., 2013). Here, our study further demonstrates that PpMYBPA1 can activate both *PpLAR* and *PpANR*, and requires a partner of PpbHLH3 but not PpbHLH33. Similar phenomenon was also observed in anthocyanin accumulation of fruits such as apple (Espley et al., 2007) and peach (Zhou et al., 2015), where MYB regulators MdMYB10 and PpMYB10.1 uses bHLH3 rather than bHLH33 as a partner to activate transcription of anthocyanin pathway genes. However, PpMYB7, unlike PpMYBPA1, can use either PpbHLH3 or PpbHLH33 as a partner. A residue at the position 72 in the bHLH binding domain shows a great variation. This residue is identical between PpMYB7 and PpMYB10.1, which differ in the bHLH selection. PpMYB10.1 is able to physically interact with both PpbHLH3 and PpbHLH33 (unpublished data). Thus, it seems that PA- or anthocyanin-related MYBs can interact with either bHLH3 or bHLH33 to form a complex, but some of the MYB-bHLH33 complexes could not directly activate flavonoid biosynthesis. In Solanaceae, MYB regulators act with JAF13 to activate the expression of AN1, resulting in the generation of a MYB/AN1 complex that induces the transcription of anthocyanin structural genes (Montefiori et al., 2015). This hierarchy of bHLHs has also been reported in other dicots such as *Arabidopsis* and Petunia (Albert et al., 2014). In peach, there are two flavonoid-related bHLH regulators, PpbHLH3 and PpbHLH33, which belong to bHLH2/AN1/TT8 clade and bHLH1/JAF13/EGL3 clade, respectively. Our recent study indicates that the expression levels of *PpbHLH3* and *PpbHLH33* are similar between white-flesh and blood-flesh peaches, whereas, the expression of *PpMYB10.1* is significantly higher in blood-flesh than in white-flesh peaches (Zhou et al., 2015). Thus, it appears that the bHLH heirarchy aspect is not conserved in peach because the *bHLH3* expression is not activated in blood peach.

Taken together, the *PpMYB7* gene represents a novel group of PA-related MYBs, which may have diverged in function from the *MYBPA1, MYBPA2, MYB5*, and *PAR* genes in plants. In peach, at least two *MYB* genes, *PpMYBPA1* and *PpMYB7*, are involved in the regulation of PA accumulation in fruits. Multiple MYBs such as *VvMYBPA1, VvMYBPA2, VvMYB5a*, and *VvMYB5b* are also reported to regulate PA accumulation in grapevine. Similarly, three *MYB* regulators, *MtPAR, MtMYB5*, and *MtMYB14* are involved in the regulation of PA accumulation in *Medicago truncatula* (Verdier et al., 2012; Liu et al., 2014). Thus, it seems that PA biosynthesis is controlled by a network of MYBs, as demonstrated by the recent finding that the MBW complex accommodates two different R2R3-MYBs to regulate PA synthesis (Albert et al., 2014; Liu et al., 2014). In addition, we checked the publicly available PLAZA database^3^ and found that the *MYB7* gene is lost in some plant species, such as *Arabidopsis, Brassica rapa*, potato, and tomato. This suggests that the complex transcriptional regulatory networks of PA biosynthesis have diverged among different plant species.

### Abscisic Acid may be Involved in the Regulation of PA Biosynthesis in Peach Fruit

Proanthocyanidins accumulate most abundantly in immature fruits such as apple (Henry-Kirk et al., 2012), bilberry (Jaakola et al., 2002), banana (Barnell and Barnell, 1945), and strawberry (Symons et al., 2012; Schaart et al., 2013), and levels decrease toward ripening In this study, a decreasing trend was also observed for flavan-3-ols in peach fruit, consistent with a previous report in nectarine (Ravaglia et al., 2013). However, few studies have been reported any mechanism underlying dynamic accumulation of PAs in fruits.

Several studies show that phytohormones such as abscisic acid (ABA) participate in the regulation of PA synthesis in fruits such as persimmon (Akagi et al., 2012) and grapevine (Lacampagne et al., 2009). In persimmon, DkbZIP5 can bind ABRE *cis*-elements in the promoter of PA-related MYB activator DkMYB4 via an ABA signal, resulting in the accumulation of PAs (Akagi et al., 2012). In this study, three ABRE *cis*-elements were identified in the promoter of *PpMYBPA1*. However, the promoter of *PpMYB7* does not contain any ABRE *cis*-elements, but has an ABA-dependent DRE2 element (Busk et al., 1997). Both *PpMYBPA1* and *PpMYB7* can be activated by PpbZIP5 via the ABA signaling. ABA biosynthesis decreases in peach fruits at late stages of development (Zhang et al., 2009). Thus, it seems that ABA may play an important role in the regulation of PA biosynthesis in peach fruit.

## Conflict of Interest Statement

The authors declare that the research was conducted in the absence of any commercial or financial relationships that could be construed as a potential conflict of interest.

## Footnotes

1 http://www.rosaceae.org/species/prunus_persica/genome_v1.0

2 http://www.dna.affrc.go.jp/PLACE/

3 http://bioinformatics.psb.ugent.be/plaza/
