# Supplementary material for: Peach MYB7 activates transcription of the proanthocyanidin pathway gene encoding leucoanthocyanidin reductase, but not anthocyanidin reductase
Source: Front Plant Sci. 2015 Oct 26;6:908. doi: 10.3389/fpls.2015.00908 (PMC4620396; doi:10.3389/fpls.2015.00908)
Supplement: Supplementary file 1 [file Data_Sheet_1.PDF]

Supplementary data

Table S1. Sequences of primers used for cloning genes and vectors construction

Primer name	Sequence	Description
PpMYB7topoF	CACCATGGCTCCAAAGAAGAATGATGG	cloning MYB7
PpMYB7topoR	CTACATAATATTGCTGACCTTTTCTCTG	
PpbHLH3topoF	CACCATGGCTGCACCGCCAAGT	cloning PpbHLH3
PpbHLH3topoR	CTAGGAATCAGATTGGGGAATTATT	
PpbHLH33topoF	CACCATGGCTAATGGGACTCAAAACCA	cloning PpbHLH33
PpbHLH33topoR	TCAACACTTACCGGCAATTTTCC	
PpMYBP7F	GGAATTCCATATGATGGCTCCAAAGAAGAATGATGG	inserting PpMYB7 into Y2H vector pGADT7 and pGBKT7
PpMYBP7R	CGCGGATCCCTACATAATATTGCTGACCTTTTCTCT	
PpMYB7NTF	GGAATTCCATATGATGGCTCCAAAGAAGAATGATGG	inserting PpMYB7 R2R3 domain (1-118) into Y2H vector pGBKT7
PpMYB7NTF	cgcggatccctaGATTTTTTTGCTCAAATGAGAATTC	
PpbHLH3NTBDF	CCGGAATTCATGGCTGCACCGCCAAGT	inserting PpbHLH3(1-235) into Y2H vector pGADT7 and pGBKT7
PpbHLH3NTBDR	CGCGGATCCCTATGGGTCGGGGCACATGG	
PpbHLH33NTBDF	GGAATTCCATATGATGGCTAATGGGACTCAAAACC	inserting PpbHLH33(1-235) into Y2H vector pGADT7 and pGBKT7
PpbHLH33NTBDR	CGCGGATCCCTATGTATCAACTATTTCATGGTCAACCT	
PpbHLH3BDF	ccggaattcATGGCTGCACCGCCAAGT	inserting PpbHLH3 into Y2H vector pGBKT7
PpbHLH3BDR	cgcggatccCTAGGAATCAGATTGGGGAATTATT	
PpbHLH33BDF	ggaattccatatgATGGCTAATGGGACTCAAAACC	primers used for inserting PpbHLH33 into Y2H vector pGBKT7
PpbHLH33BDR	cgcggatccTCAACACTTACCGGCAATTTTCC	
PpMYBP7NLUCF	CGGGGTACCATGGCTCCAAAGAAGAATGATGG	primers used for inserting PpMYB7 into vector pCambia1300-NLuc
PpMYBP7NLUCR	ACGCGTCGACCATAATATTGCTGACCTTTTCTCTGT	
PpbHLH3CLUCF	CGCGGATCCCGCTGCACCGCCAAGTAGC	primers used for inserting PpbHLH3 into vector pCambia1300-CLuc
PpbHLH3CLUCR	ACGCGTCGACCTAGGAATCAGATTGGGGAATTATT	
PpbHLH33CLUCF	CGCGGATCCCGCTAATGGGACTCAAAACCATG	primers used for inserting PpbHLH33 into vector pCambia1300-CLuc
PpbHLH33CLUCR	ACGCGTCGACTCAACACTTACCGGCAATTTTCC	
PpMYBPA1OEF	CCCAAGCTTATGGGAAGGGCTCCTTGTTG	Primers used for inserting PpMYBPA1 into vector pSAK277
PpMYBPA1OER	GCTCTAGATTATATCAGCAGTGACTCAGCAAATG	
PpbZIP5OEF	CCGGAATTCATGGGGTTTCAGACTATGGCTTC	Primers used for inserting PpbZIP5 into vector pSAK277
PpbZIP5OER	CCGCTCGAGTCAGAAAAAGGCTGAACTTATTCTTC	
PpbHLH3ADF	CCGGAATTCATGGCTGCACCGCCAAGT	Primers used for inserting PpbHLH3 into vector pGADT7
PpbHLH3ADR	CGCGGATCCCTAGGAATCAGATTGGGGAATTATT	
PpbHLH33ADF	GGAATTCCATATGATGGCTAATGGGACTCAAAACC	Primers used for inserting PpbHLH33 into vector pGADT7
PpbHLH33ADR	CGCGGATCCTCAACACTTACCGGCAATTTTCC	
proLAR1-LUCF	CGCGGATCCGGTGCTGATGATCAATGACTGC	Primers used for inserting proPpLAR1 into vector pGreenII LUC+
proLAR1-LUCR	CATGCCATGGCTGGCTGCTGGCT	
proANR-LUCF	CCCCCCGGGCGCTGTTATGGAAAGGGTCACT	Primers used for inserting proPpANR into vector pGreenII LUC+
proANR-LUCR	GCTGTCTTCTTTGAGATGGGTTG	
proDFR-LUCF	CGCGGATCCGAATGCACTACTGGAACCGACTG	Primers used for inserting proPpDFR into vector pGreenII LUC+
proDFR-LUCR	CATGCCATGGTTGAATCAAATCAAGTATGTAC	
proUFGTF	CTGTGCCGCAATATCTGACATC	Primers used for cloning proPpUFGT
ProUFGTR	TATGAGCTAATAAGACTAATTGGAGTGG	

Table S2. Sequences of primers used for qRT-PCR analysis in peach

Gene	Gene number	Forward (5’-3’)	Reverse (5’-3’)
PpLAR1	ppa007994m	CTATACGACATCAATGGTCTGGC	TTCTGGTATGCGGTTCTCTGC
PpLAR2	ppa024512m	GCAACCTCTTCAGCATCAACC	GTGGGATTCGCATTTCTTCAG
PpANR	ppa008295m	TCTCATCACAGTCATCCCTTCTC	CAAGGCATGATTTATGAGGAAGT
PpMYBPA1	ppa009439m	GGGAAGGCCATTGGAGATC	AACGGTTGCCCAGAAGTGA
PpMYB7	ppa016135m	TTCCATAGCAGGTTTGAATCG	TTCATCATCAGAAATGTTGCCTC
PpTEF2	ppa001368m	GGTGTGACGATGAAGAGTGATG	TGAAGGAGAGGGAAGGTGAAAG

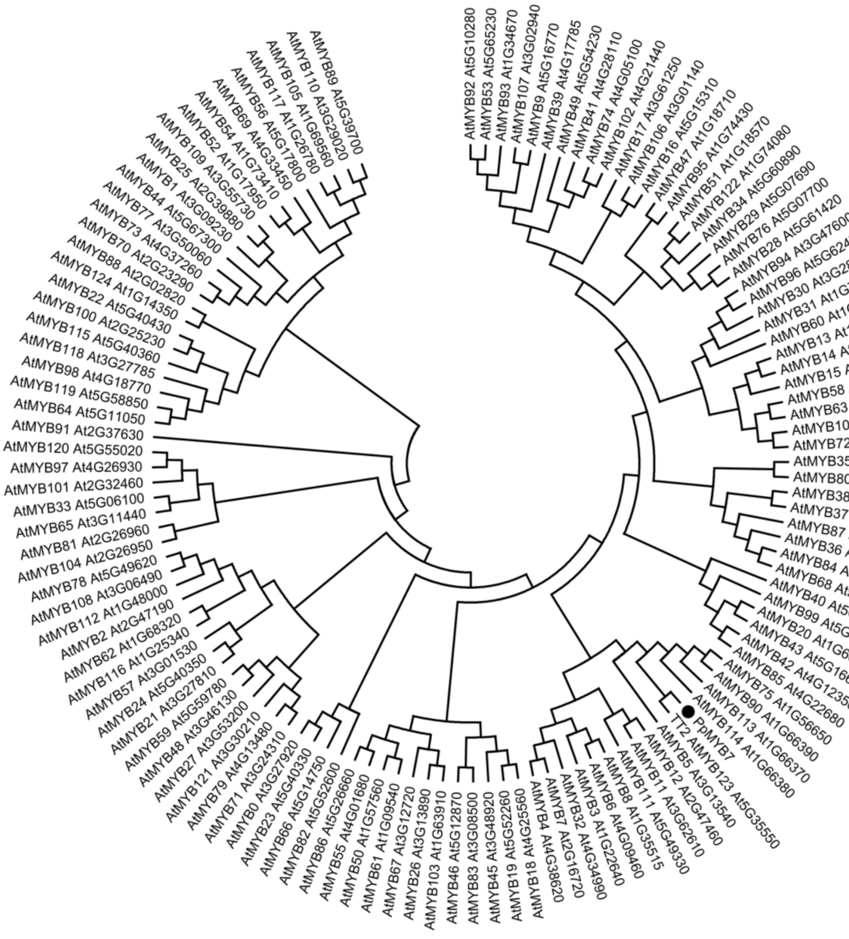

Figure S1 Phylogenetic tree derived from global amino acid sequences of genes encoding R2R3-MYB transcription factors in *Arabidopsis thaliana* and *PpMYB7*. The *PpMYB7* gene isolated in this study is highlighted with a black circle. The full length amino acid sequences were aligned using Muscle software. Phylogenetic tree was conducted using MEGA version 6.0 using Minimum evolution method and 1,000 bootstrap replicates.The scale bar represents 0.1 substitutions per site. All the sequences in *Arabidopsis thaliana* were retrieved from TAIR website (https://www.arabidopsis.org/).

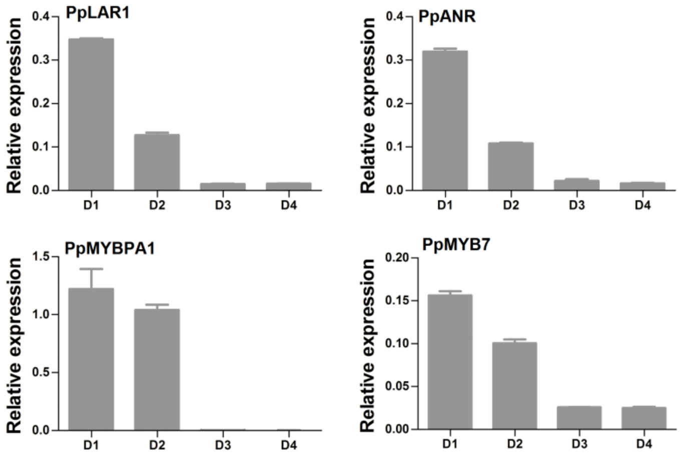

Figure S2 qRT-PCR analysis of the expression profiles of genes related to PA synthesis and flavan-3-ol content in fruits of peach cv. Baifeng that were at different stages of development in 2013. The results represent the means of three technical replicates. D1 to D4 indicate 30, 64, 85, and 92 DAFB, respectively. Error bars show SE of the mean.

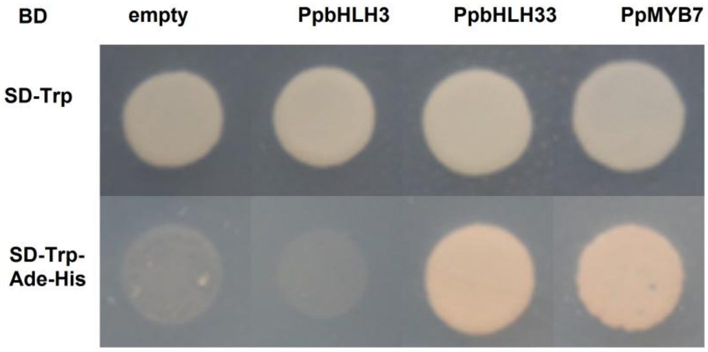

Figure S3 Autoactivation test for PpMYB7 and PpbHLHs in yeast. Full length of PpMYB7, PpbHLH3 and PpbHLH33 was fused with pGBKT7 vector and transferred into yeast strain ‘Y2Hgold’, and empty vector was used as negative control. Photographs were taken 3 days after incubation.

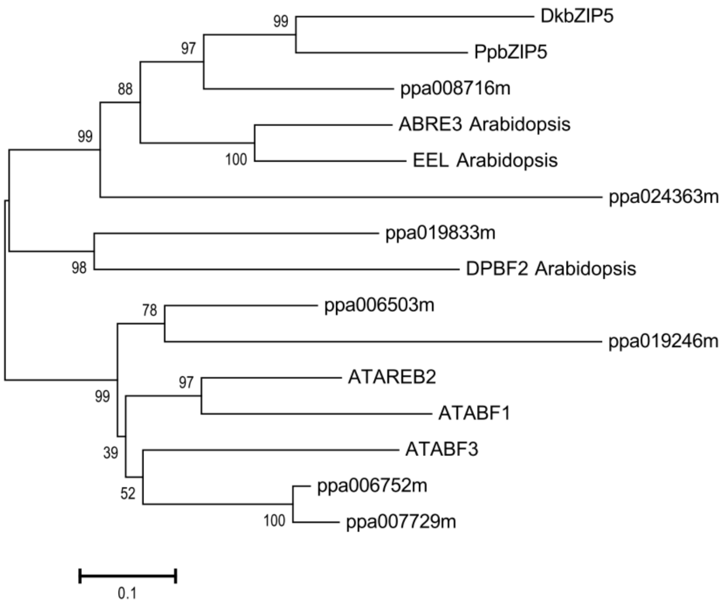

Figure S4 Phylogenetic tree derived from global amino acid sequences of genes encodingABF-, AREB-, and ABI5-Like bZIP transcription factors in *Arabidopsis thaliana* and *Prunus persica*. The full length amino acid sequences were aligned using Muscle software. Phylogenetic tree was conducted using MEGA version 6.0 using neighbor joining method and 1,000 bootstrap replicates. The scale bar represents 0.1 substitutions per site. All the sequences in *Arabidopsis thaliana* were retrieved from TAIR website (https://www.arabidopsis.org/), and in *Prunus persica* from GDR database (https://www.rosaceae.org/node/1).
